# Landscape structure affects the prevalence and distribution of a tick-borne zoonotic pathogen

**DOI:** 10.1186/s13071-018-3200-2

**Published:** 2018-12-04

**Authors:** Caroline Millins, Eleanor R. Dickinson, Petra Isakovic, Lucy Gilbert, Agnieszka Wojciechowska, Victoria Paterson, Feng Tao, Martin Jahn, Elizabeth Kilbride, Richard Birtles, Paul Johnson, Roman Biek

**Affiliations:** 1 Institute of Biodiversity, Animal Health and Comparative Medicine, University of Glasgow, Glasgow, Scotland, UK; 2 The Boyd Orr Centre for Population and Ecosystem Health, University of Glasgow, Glasgow, Scotland, UK; 3 School of Veterinary Medicine, University of Glasgow, Glasgow, Scotland, UK; 4 Present address: Zakot 43, 8250 Brezice, Slovenia; 5 James Hutton Institute, Craigiebuckler, Aberdeen, Scotland, UK; 6 Present address: Faculty of Biology and Environmental Protection, University of Lodz, Lodz, Poland; 7 Present address: Wayne State University, 42 W. Warren Ave, Detroit, MI 48202 USA; 8 Present address: GEOMAR - Helmholtz Centre for Ocean Research Kiel, Düsternbrooker Weg 20, D-24105 Kiel, Germany; 9 School of Environment and Life Sciences, University of Salford, Salford, England, UK

**Keywords:** Habitat fragmentation, Pathogen persistence, Host community, *Borrelia burgdorferi* (*sensu lato*)

## Abstract

**Background:**

Landscape structure can affect pathogen prevalence and persistence with consequences for human and animal health. Few studies have examined how reservoir host species traits may interact with landscape structure to alter pathogen communities and dynamics. Using a landscape of islands and mainland sites we investigated how natural landscape fragmentation affects the prevalence and persistence of the zoonotic tick-borne pathogen complex *Borrelia burgdorferi* (*sensu lato*), which causes Lyme borreliosis. We hypothesized that the prevalence of *B. burgdorferi* (*s.l.*) would be lower on islands compared to the mainland and *B. afzelii*, a small mammal specialist genospecies, would be more affected by isolation than bird-associated *B. garinii* and *B. valaisiana* and the generalist *B. burgdorferi* (*sensu stricto*).

**Methods:**

Questing (host-seeking) nymphal *I. ricinus* ticks (*n* = 6567) were collected from 12 island and 6 mainland sites in 2011, 2013 and 2015 and tested for *B. burgdorferi* (*s.l*.). Deer abundance was estimated using dung transects.

**Results:**

The prevalence of *B. burgdorferi* (*s.l.*) was significantly higher on the mainland (2.5%, 47/1891) compared to island sites (0.9%, 44/4673) (*P* < 0.01). While all four genospecies of *B. burgdorferi* (*s.l.*) were detected on the mainland, bird-associated species *B. garinii* and *B. valaisiana* and the generalist genospecies *B. burgdorferi* (*s.s.*) predominated on islands.

**Conclusion:**

We found that landscape structure influenced the prevalence of a zoonotic pathogen, with a lower prevalence detected among island sites compared to the mainland. This was mainly due to the significantly lower prevalence of small mammal-associated *B. afzelii*. Deer abundance was not related to pathogen prevalence, suggesting that the structure and dynamics of the reservoir host community underpins the observed prevalence patterns, with the higher mobility of bird hosts compared to small mammal hosts leading to a relative predominance of the bird-associated genospecies *B. garinii* and generalist genospecies *B. burgdorferi* (*s.s.*) on islands. In contrast, the lower prevalence of *B. afzelii* on islands may be due to small mammal populations there exhibiting lower densities, less immigration and stronger population fluctuations. This study suggests that landscape fragmentation can influence the prevalence of a zoonotic pathogen, dependent on the biology of the reservoir host.

**Electronic supplementary material:**

The online version of this article (10.1186/s13071-018-3200-2) contains supplementary material, which is available to authorized users.

## Background

Landscape structure can influence the persistence of a pathogen by affecting the movement of hosts and vectors, and thus the potential for transmission. As host populations become reduced within smaller habitat patches, stochastic fadeout of transmission is expected to become more common. Pathogen persistence will then depend on the degree of isolation of habitat patches, and a balance between colonization and extinction events [1, 2]. Different biological processes are predicted to affect colonization and extinction events. For example, colonization of a pathogen into new habitat patches is likely to be affected by the mobility and ecology of the host species. In contrast, host population dynamics, particularly large seasonal or interannual fluctuations, can contribute to stochastic fadeout of a pathogen within patches [3].

Environmental change can affect disease risk to humans or livestock, with vector-borne pathogens especially sensitive to changes in habitat and climate [4]. Following historic reduction and fragmentation of woodlands in Europe, there are now widespread efforts to increase the amount of woodland, as well as connectivity between patches, to promote biodiversity and restore ecological processes [5]. This has the potential to affect the distribution and prevalence of vector-borne pathogens associated with forest ecosystems. In general, woodlands are favourable habitats for the tick vector *Ixodes ricinus*, as they support higher densities of hosts that can provide blood meals for the tick compared to open habitats, and the ground leaf litter layer provides humid conditions for off-host tick development and survival [6]. *Ixodes ricinus* is a generalist tick vector that transmits many pathogens of importance for human and animal health, including the bacteria of the *Borrelia burgdorferi* (*sensu lato*) (*s.l.*) complex, which are the cause of Lyme borreliosis, an emerging disease in the northern hemisphere [7, 8].

Host community composition is an important factor determining persistence of tick-borne pathogens including *B. burgdorferi* (*s.l.*) and one mechanism by which environmental change can affect disease risk [9–12]. While many vertebrate species provide blood meals for the tick *I. ricinus*, not all species are competent to transmit *B. burgdorferi* (*s.l.*) At least nine genospecies of *B. burgdorferi* (*s.l.*) with differing pathogenicities, clinical signs and reservoir host associations are known to be circulating in Europe [13, 14]. Three of these together, *B. afzelii* which is adapted to small mammals [15], *B. garinii* which is adapted to bird reservoir hosts [16] and *B. burgdorferi* (*s.s.*), a generalist genospecies, are collectively responsible for most cases of human disease [17]. Deer are generally considered non-competent hosts for genospecies of *B. burgdorferi* (*s.l.*) and feed all life-stages of the tick *I. ricinus* [18–20]. As deer feed significant numbers of adult female ticks which go onto produce immature larval stages they are sometimes termed ‘tick reproduction hosts’ [21]. Deer density is frequently positively linked with nymphal tick density, e.g. [22, 23]; however no significant relationship has also been found in other studies [24]. As incompetent hosts for *B. burgdorferi* (*s.l.*) and as hosts of ticks, deer can affect transmission, with both amplification and dilution of transmission predicted from theoretical models depending on how tick vectors are distributed amongst the host community [25].

Little is known about the effects of landscape structure and fragmentation on the risk of Lyme borreliosis in Europe. Previous studies suggested that key small mammal reservoir hosts such as bank voles (*Myodes glareolus*) may decrease in density in fragmented woodland [26, 27]. This could reduce persistence of small mammal-associated genospecies of *B. burgdorferi* (*s.l.*) within isolated patches of habitat due to stochastic extinction events. Also, as birds are unable to efficiently transmit small mammal-associated *B. afzelii*, this limits the reintroduction of this pathogen among suitable areas of habitat in fragmented landscapes [28]. Therefore, we expect landscape fragmentation to have different effects on the prevalence and persistence of the genospecies of *B. burgdorferi* (*s.l.*) in Europe, with small mammal-associated *B. afzelii* being less likely to persist and present at lower prevalence in fragmented landscapes compared to bird-associated and generalist genospecies.

Here we use a natural island system located in a large freshwater lake in Scotland as a model to investigate the effect of landscape fragmentation on *B. burgdorferi* (*s.l.*) prevalence. Four genospecies of *B. burgdorferi* (*s.l.*) are known to occur in Scotland, *B. afzelii*, *B. garinii* and *B. burgdorferi* (*s.s*.) are present and also *B. valaisiana* which is associated with birds and is uncommonly associated with human disease. The two species of small mammal which are widely distributed and present at highest abundance in woodland habitats are the bank vole and the wood mouse (*Apodemus sylvaticus*). Other mammal species that may be significant reservoir hosts in the area include the grey squirrel (*Sciurus carolinensis*) [29], the red squirrel (*Sciurus vulgaris*) [30] and the common (*Sorex araneus*) and pygmy shrews (*Sorex minutus*) [30]. Passerine birds are also hosts for ticks in UK woodlands [31], with particularly high tick burdens on ground foraging species such as the common blackbird (*Turdus merula*) and the song thrush (*Turdus philomelos*). Fallow deer (*Dama dama*) are present in the study area.

We first hypothesized that islands would be less likely to support endemic circulation of genospecies relying on small mammal hosts compared to continuous mainland habitat. We predicted that islands would have a lower prevalence of *B. afzelii*, due to existing evidence suggesting that there are lower densities of small mammals in fragmented habitat [26, 27] and that islands experience local extinction of *B. afzelii* more frequently. In contrast, no such restriction is expected for bird specialist and generalist genospecies since birds traveling between the mainland and islands can reintroduce infected ticks following local extinctions. Secondly, we hypothesized that there would be decreasing likelihood of *B. burgdorferi* (*s.l.*) presence with increasing isolation from the mainland, due to less frequent movement between the mainland and isolated habitat patches and reduced opportunities for recolonization. According to this, smaller and more distant islands are predicted to be less likely to support pathogen persistence due to lower reservoir host density and increased effects of stochastic events. From this we predict that (i) islands overall have a lower *B. burgdorferi* (*s.l.*) prevalence than mainland sites; (ii) *B. garinii* would be more prevalent than *B. afzelii* on islands due to greater dispersal by birds compared to small mammals; and (iii) smaller more isolated islands would have a lower prevalence than larger islands located closer to the mainland.

## Methods

### Study sites

**Fig. 1:**
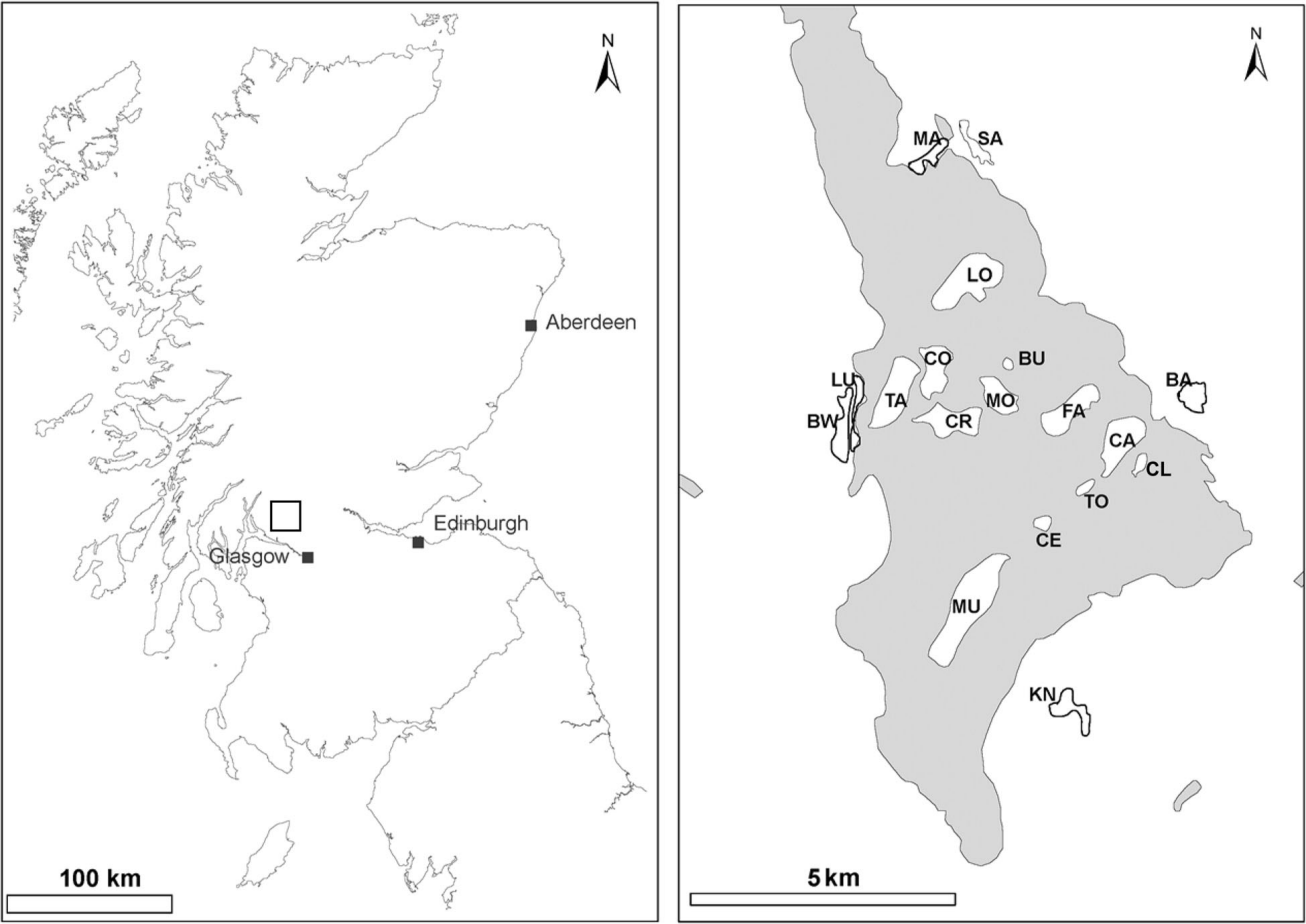
Map of the study sites within Loch Lomond and the Trossachs National Park, Scotland, is shown by the box on the map on the left side of the figure. Loch Lomond is represented by the shaded area on the map on the right, the 12 islands within the loch which were sampled for questing *Ixodes ricinus* and 6 mainland sites are labelled. Site descriptions are in Table 1. Maps were drawn in ArcMap, ArcGIS version 10 (Esri, Redlands, USA)

To investigate *I. ricinus* abundance, *B. burgdorferi* (*s.l.*) prevalence, and environmental and host associations in relation to landscape fragmentation, we sampled from among six mainland sites and twelve islands sites in 2011, 2013 and 2015 within Loch Lomond and Trossachs National Park (Fig. 1, Table 1). Sampling was conducted every other year to enable patterns of pathogen prevalence and persistence to be studied over an extended temporal time frame. The park is in the west of Scotland and contains the largest natural freshwater area in the United Kingdom. The islands are situated within the southern part of this freshwater lake. The Loch Lomond shoreline is surrounded by mature oak (*Quercus* spp.) and birch (*Betula* spp.) woodland with areas of managed coniferous forest. The ground vegetation is a mixture of grasses, mosses, bracken (*Pteridium aquilinum*), bilberry (*Vaccinium myrtillus*), bramble (*Rubus fruticosus*), and ericaceous species (*Calluna vulgaris* and *Rhododendron* spp.). The islands sampled within Loch Lomond varied in size from 0.03 km^2^ to 1.2 km^2^ and are situated between 0.3 km and 2.4 km from the mainland (Table 1). The predominant woodland cover on the islands, like the mainland sites, is also oak and birch woodland. Mainland sites were chosen to have similar habitat characteristics to the islands, and were located within 0.5 km of the Loch shoreline. Both island and mainland sites were known prior to this study to have populations of *I. ricinus*, fallow deer (*Dama dama*), and other woodland mammal and bird species typical of the area (Additional file 1: Table S1) [27].

**Table 1:** Study sites on Loch Lomond mainland and islands, with vegetation, two indices of deer density based on deer counts and deer dung, and island size and distance to the mainland

Site	Site type	Vegetation^a^	Mean deer density (per km^2^)^b^	Deer density (dung transects)^c^	Island area (km^2^)	Distance to the mainland (km)
BA	Mainland	Scots pine, oak and birch, understorey of grass, moss and bracken.	na	17.3	na	na
BW	Mainland	Oak with understorey of dense areas of bramble and bracken.	na	5.9	na	na
MA	Mainland	Oak with mixed understory of blueberry, grass and moss	na	1.1	na	na
KN	Mainland	Oak with birch and holly. Understorey relatively open with blueberry, bramble and grasses.	na	0.9	na	na
LU	Mainland	Oak with understorey of dense areas of bramble and bracken.	na	na	na	na
SA	Mainland	Oak/managed pine with understorey of bramble, blueberry and grasses	na	8.7	na	na
BU	Island	Scots pine, birch and oak. Understorey of rhododendrons, bracken and grass species	0.0	17.5	0.031	1.9
CA	Island	Oak, birch, holly and alder. Understorey dense with bracken and grass species.	68.9	5.0	0.53	0.28
CE	Island	Oak, relatively open understorey with blueberry and bracken.	157.9	0.0	0.056	1.7
CL	Island	Oak, grass and bracken understorey.	0.0	2.2	0.056	0.86
CO	Island	Oak with blueberry and moss understorey	2.4	3.4	0.42	1.7
CR	Island	Birch, oak and alder. Understoreys relatively open with blueberry, bracken and moss species.	28.3	33.3	0.28	2.4
FA	Island	Oak, with a sparse understorey of bracken and bramble.	130.9	28.2	0.45	0.59
LO	Island	Oak, birch, and yew trees. Understorey dense with bracken.	43.3	31.1	0.75	0.77
MO	Island	Birch and alder. Understorey of rhododendrons, blueberry, gorse and bog myrtle.	26.3	22.3	0.46	1.2
MU	Island	Oak, birch and Scots pine. An understorey of blueberry, bracken and bramble.	0	3.1	1.2	1.1
TA	Island	Oak with blueberry and moss understorey	28.1	32.7	0.63	0.31
TO	Island	Oak and birch. Understorey dense with bluberry and bracken.	26.7	32.1	0.075	1.9

^a^Vegetation data recorded 2009–2010 [27]

^b^Mean deer density from deer count data collected by Scottish National Heritage counts on Loch Lomond islands in March 2008 and March 2012

^c^Deer density estimated from dung counts along transects, May-July 2016

### Field collection of ticks

Questing ticks were collected from the vegetation by blanket dragging during the peak tick questing period, between April and August. Blanket dragging was carried out between 10:00 and 16:00 h, when the vegetation was dry or at least two hours after rainfall. Ticks were collected by dragging a white blanket slowly across the surface of the vegetation until 200 nymphs were collected, or for a minimum of 3 h per site in order to collect a representative sample of ticks to estimate the prevalence of *B. burgdorferi* (*s.l.*). Nymphs attached to the blanket were counted and stored in 70% ethanol after each drag. Standardized drags to estimate nymph density were also carried out, the methodology and results are provided in Additional file 1: Text S2 and Table S3. We focussed on nymphs as they are considered to be the most important life-stage in *B. burgdorferi* (*s.l.*) transmission to humans [32].

### Host and environmental predictors of tick abundance and *B. burgdorferi* (*s.l.*) prevalence

Both island and mainland sites were known prior to the study to have populations of *I. ricinus* and woodland mammals and birds typical of the area [27] (Additional file 1: Table S1). While it was not practical to estimate the density of all potential bird and small mammal reservoir hosts across the multiple sites and years of this study, deer density was estimated. As both amplification and dilution effects of deer density on *B. burgdorferi* (*s.l.*) prevalence have been predicted from theoretical models [25], this was considered an important parameter to include in statistical models to investigate the effect of landscape fragmentation on *B. burgdorferi* (*s.l.*) prevalence [25]. Deer density was quantified on both island and mainland sites in 2015 using a deer dung transect method ([33], Additional file 1: Text S1, Table S2). There was no significant difference between the mean deer density estimated on island and mainland sites (Kruskal-Wallis *χ*^2^ = 0.11, *df* = 1, *P* = 0.74). Data from two deer count surveys of the Loch Lomond Islands, carried out by park staff in 2008 and 2012 (Additional file 1: Text S1, Table S2) indicated that deer density remained consistent through time (Spearman’s rank correlation coefficient = 0.65, *P* = 0.02). There was also no difference between the mean deer density estimated in the deer surveys in 2008, 2012 and the deer dung survey in 2015 (Kruskal-Wallis *χ*^2^ = 0.78, *df* = 2, *P* = 0.68). Therefore, there was strong reason to assume that densities estimated from the deer dung transect survey as the only method for which data from both islands and mainland were available, could reasonably be used as a measure of deer density across all years of this study.

### DNA extraction, *B. burgdorferi* (*s.l.*) detection and genospecies determination

Tick DNA extraction, PCR preparation and genospecies determination using PCR products were carried out in separate laboratories. Each nymph was placed in a separate Eppendorf tube and DNA extracted using a basic ammonia extraction technique [34]. One DNA extraction control which included reagents but no tick was included with every 11 samples. Detection of *B. burgdorferi* (*s.l.*) was either by a nested PCR [35] which targets the 5S-23S intergenic spacer (IGS) region or by a real time PCR [36] which detects a fragment of the *23S* ribosomal RNA gene. The PCR protocol and cycling conditions were followed as previously described [36]. A negative and a positive control were included in each PCR run. Genospecies determination was carried out on all PCR-positive nymphs, either by reverse line blotting as previously described [37, 38] and/or by sequencing the PCR product from the 5S-23S intergenic spacer region [39]. Where sequencing was performed, PCR products were sequenced with the forward and reverse primers using Sanger sequencing. Sequences were trimmed in Geneious version 7.0.6 (Biomatters Ltd). To determine the genospecies each sequenced and trimmed IGS PCR product was subjected to a BLAST search against the National Centre for Biotechnology Nucleotide BLAST database. Sequences were also examined for polymorphisms characteristic of each genospecies as described previously [40].

### Statistical analysis

All statistical analyses were carried out in R version 3.4.1 [41]. We calculated the probability *p* of not detecting *B. burgdorferi* (*s.l.*) given a sample of *n* tested nymphs and an expected prevalence *P* as

$$ p={\left(1-P\right)}^n $$

All GLMMs were fitted using the *lme4* package [42], starting with a maximal global model, which included all fixed effects and interaction terms where specified. Model selection was performed by backward stepwise elimination, based on minimising the Akaike information criterion (AICc) [43, 44]. The *AICcmodavg* package [44] was used to calculate the second-order Akaike’s information criterion (AICc) to account for small sample size. The delta AICc, defined as the rise in AICc after removing each variable from the selected model was calculated for each explanatory variable in turn. We gauged the explanatory power of the models by partitioning the total variance into three components: variation explained by the fixed effects; unexplained variation between sites; and unexplained variation between observations/drags within sites. The proportion of the sum of these three variances explained by the fixed effects is defined as the latent-scale marginal R^2^, R^2^_GLMM(m)*_[45]. The 95% confidence intervals (CI) for proportions were calculated using the prop.test( ) function in ‘R’.

#### Testing for association between habitat fragmentation and *B. burgdorferi *(*s.l*.) prevalence

*B. burgdorferi* (*s.l.*) infection in nymphs (infected or uninfected) was modelled with a logit-binomial general linear mixed model (GLMM) as a function of the following explanatory variables: fragmentation class (island or mainland), year (2011, 2013 or 2015), deer density (estimated from dung transects) and an interaction between fragmentation class (island/mainland) and year. To control for repeated sampling of sites and overdispersion, random effects of sampling site and observation [46] were included. This model was repeated with the individual genospecies *B. afzelii*, *B. garinii* and *B. burgdorferi* (*s.s.*) infection in nymphs (infected or not infected) as the outcome variable. There were insufficient data to repeat the model with *B. valaisiana*. To test if effects of landscape structure on *B. burgdorferi* (*s.l.*) prevalence were a result of differences in *B. afzelii* prevalence, the model was repeated with infected nymphs of the three genospecies: *B. garinii*, *B. burgdorferi* (*s.s.*) and *B. valaisiana* as the outcome variable. As deer density was not maintained in the models during model selection, they were re-run with data from all site-year combinations (*n* = 42 compared to *n* = 40, as one site did not have a deer density estimate).

#### Testing for associations between island size, island isolation and *B. burgdorferi *(*s.l*.) prevalence

Data collected from all island sites in 2011, 2013 and 2015 were used for this model. *Borrelia burgdorferi* (*s.l.*) infection in nymphs (infected or uninfected) was modelled with a logit-binomial GLMM as a function of the following explanatory variables: island size, distance to the mainland, deer density and year with random effects of site and observation. There were insufficient data to model individual genospecies separately.

## Results

**Fig. 2:**
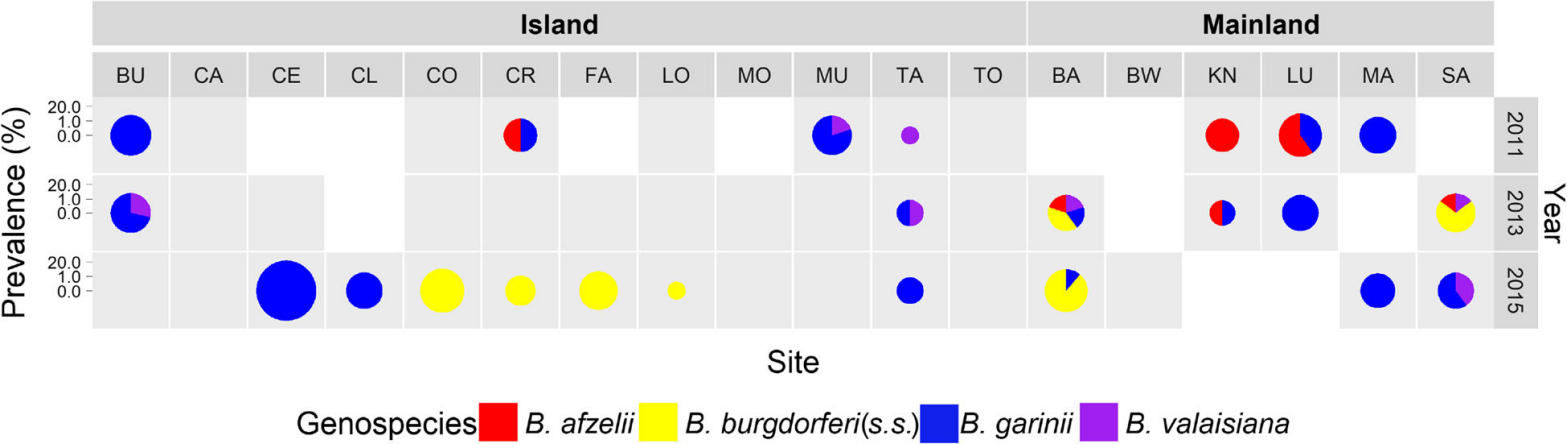
Prevalence and genospecies of *Borrelia burgdorferi* (*sensu lato*) sampled at Loch Lomond and the Trossachs National Park, Scotland in 2011, 2013 and 2015. Grey squares indicate sites which were sampled, white squares indicate sites which were not sampled. The size of the circle is proportional to the log-prevalence of *B. burgdorferi* (*s.l.*), the colour indicates the genospecies present

A total of 6567 tick nymphs were collected across all sites. The overall mean prevalence of *B. burgdorferi* (*s.l.*) in questing nymphs was 1.4% (91/6567; 95% CI: 1.1–1.7%; range 0–21.2%) (Additional file 1: Table S3). The mean prevalence in questing nymphs on the six mainland sites was 2.5% (47/1891; 95% CI: 1.9–3.3%; range 0–4.6%). The prevalence in questing nymphs on the twelve islands was 0.9% (44/4673; 95% CI: 0.7–1.2%; range 0–21.2%). All four genospecies of *B. burgdorferi* (*s.l.*) which are known to occur in the UK [24, 47] were detected at mainland and island sites. Of the 47 mainland nymphs that tested positive for *B. burgdorferi* (*s.l.*) 40.4% (19/47) were infected with *B. garinii*, 31.9% (15/47) with *B. burgdorferi* (*s.s.*), 19.1% (9/47) with *B. afzelii*, and 8.5% (4/47) with *B. valaisiana.* On island sites, of the 44 nymphs that tested positive for *B. burgdorferi* (*s.l.*), 63.6% (28/44) were infected with *B. garinii*, 22.7% (10/44) with *B. burgdorferi* (*s.s.*), 11.4% with *B. valaisiana* (5/44) and 2.3% with *B. afzelii* (1/44) (Fig. 2, Additional file 1: Table S3).

There was variation in genospecies distribution over the study period, with many apparent introductions and losses of genospecies from individual sites (Fig. 2). Whereas 10 out of 11 mainland site collections included infective nymphs, this was only true for 13 out of 31 collections on the islands. When comparing only collections in which infected nymphs were detected, the prevalence on island and mainland sites was comparable (2.4% and 2.75%). Using our overall prevalence estimate for the mainland as the expected prevalence, we calculated the probability of not detecting any infected ticks in our sample for each of the island site collections. For the majority of island site collections without *B. burgdorferi* (*s.l.*) detection (14 out of 18, Additional file 1: Table S3), the probability of not detecting the pathogen if it was present was low (< 0.03). For the remaining 4 collections, the probability exceeded 0.05, indicating that absence of the pathogen could not be inferred with confidence for these collections due to limited sample size.

### *Borrelia burgdorferi* (*s.l.*) prevalence on island and mainland sites

Data from all sites and years were used to model *B. burgdorferi* (*s.l.*) prevalence in questing nymphs (*n* = 42 site/year sampling events). The best-fit model for *B. burgdorferi* (*s.l.*) prevalence included year and fragmentation class (island/mainland) (Table 2), an interaction between these variables was not supported. The proportion of variance explained by the fixed effects R^2^_GLMM(m)*_ was 34% while the site level random effect accounted for only 3.4% (unexplained variation between sites), and the remaining 62.6% was attributed to the observation level random effect (unexplained variation within sites). Mainland sites were significantly more likely to harbour infected nymphs compared to islands, odds ratio (OR) = 4.8 (95% CI: 1.5–15.5) (delta AICc = 3.4). Year of sampling did not contribute significantly to explaining variation in prevalence (delta AICc for year = 0.75).

**Table 2:** Best-fit models explaining variation in *Borrelia burgdorferi* (*s.l.*), *B. afzelii* and *B. garinii* prevalence in questing nymphs. Data from all sites and years are included, no measure of deer abundance is included. Results for each of the best-fit models are shown in each case. Delta AICc indicates the change in AICc after removing each variable from the best-fit model

Model description	Fixed effects	Mean (Estd)	SE	*P*-value	Delta AICc
*B. burgdorferi* (*s.l.*) prevalence	Intercept	-5.1	0.55	< 2 × 10^-16^	–
	Location (Mainland *vs* Island)	1.6	0.6	0.0074	3.4
	Year (2013 *vs* 2011)	-1.1	0.65	0.10	0.75
	Year (2015 *vs* 2011)	0.38	0.61	0.54	
Model to predict *B. afzelii* prevalence	Intercept	-9.1	1.2	1.2 × 10^-13^	–
	Location (Mainland *vs* Island)	3.2	1.2	0.0082	5.0
Model to predict *B. garinii* prevalence	Intercept	-6.1	0.59	< 2 × 10^-16^	–
Model to predict *B. burgdorferi* (*s.s*.) prevalence	Intercept	-10.8	1.8	4.2 × 10^-9^	–
Model to predict combined *B. burgdorferi* (*s.s*.), *B. garinii* and *B. valaisiana* prevalence	Intercept	5.1	0.38	< 2 × 10^-16^	–

When considering the effect of island size, distance to the mainland, deer density and year of sampling on *B. burgdorferi* (*s.l.*) prevalence, the best-fit model was the intercept only model.

### *Borrelia burgdorferi* (*s.l.*) genospecies prevalence on island and mainland sites

The best-fit model for *B. afzelii* prevalence included a fixed effect of fragmentation class (island/mainland) (Table 2). Mainland sites were significantly more likely to harbour *B. afzelii* infected nymphs compared to islands, OR = 22.5 (95% CI: 2.3–238.2) (delta AICc = 5.0). The proportion of variance explained by the fixed effect R^2^_GLMM(m)*_ was 54% with all the remaining variation accounted for by the observation level random effect. The best-fit model for *B. garinii* and *B. burgdorferi* (*s.s.*) prevalence did not include a significant effect of year or fragmentation class (island/mainland) (Table 2). The best-fit model for *B. burgdorferi* (*s.l.*) prevalence with *B. afzelii* infected nymphs removed (combined *B. garinii*, *B. burgdorferi* (*s.s.*) and *B. valaisiana* prevalence) also did not include a significant effect of year or fragmentation class (island/mainland).

## Discussion

This study investigated whether landscape fragmentation (islands *versus* mainland) affects the distribution and persistence of the tick-borne pathogen *B. burgdorferi* (*s.l.*) and three genospecies *B. afzelii*, *B. garinii* and *B. burgdorferi* (*s.s.*). Consistent with our prediction for fragmented landscapes, we found a significantly lower prevalence of *B. burgdorferi* (*s.l.*) among island sites compared to mainland sites, due to an absence of *B. burgdorferi* (*s.l.*) from over half of the sample collections at island sites. The prevalence of *B. burgdorferi* (*s.l.*) on islands with infected ticks was similar to that on the mainland, suggesting that islands were capable of supporting similar levels of transmission as the mainland. For most island site collections, the sampling effort was sufficient to detect the pathogen with reasonable confidence, had it been present. This suggests local extinctions could contribute to the high proportion of apparent absences and the low overall prevalence of *B. burgdorferi* (*s.l.*) on the islands. In general, only the bird-associated genospecies *B. garinii* and *B. valaisiana* or the generalist genospecies *B. burgdorferi* (*s.s.*) were found on islands, though their detection there was also more sporadic than on mainland sites.

As expected, the prevalence and frequency of detection of small mammal-associated *B. afzelii* was significantly lower in fragmented habitat. There are several ecological factors which may affect the persistence of *B. afzelii* on islands. A previous study reported lower densities of competent small mammal reservoir hosts on the islands compared to the mainland [27]. Low rodent densities might reduce the proportion of blood meals on competent *versus* non-competent transmission hosts below a threshold necessary for pathogen persistence [25]. In addition, seasonal and inter-annual population fluctuations, which are common in small mammal populations, could lead to stochastic extinction of the pathogen. While these effects might also affect *B. afzelii* dynamics on mainland sites, their isolated nature makes island sites less likely to be recolonised by infected rodent hosts.

In contrast, the patterns for bird-associated *B. garinii* and *B. valaisiana* and generalist genospecies *B. burgdorferi* (*s.s.*) suggested more frequent introductions to islands, likely reflecting the mobility of the bird hosts of these genospecies. However, the high mobility of bird hosts did not necessarily result in pathogen persistence on islands, and despite recording similar communities of competent bird reservoir hosts (Additional file 1: Table S1), bird-associated or generalist genospecies of *B. burgdorferi* (*s.l.*) were not detected on three islands (CA, MO, TO) and were absent from other islands during some years (Fig. 2).

Questing nymph densities were not significantly different between island and mainland sites (Additional file 1: Text S2, Table S4) and were, in general, equivalent or higher than elsewhere in Scotland where *B. burgdorferi* (*s.l.*) transmission has been detected (e.g. 0.6–11.5 nymphs/10 m^2,^ [24, 47]). Previous work in our study system had found higher levels of tick infestation on rodents on islands compared to mainland sites [27]. Therefore, vector abundance was not the limiting factor for *B. burgdorferi* (*s.l.*) presence on islands [48]. Likewise deer density which had been found to influence *B. burgdorferi* (*s.l.*) prevalence in some studies (e.g. [25, 47]) had no detectable effect in our study (Table 2). Instead, we suggest that the observed variability in *B. burgdorferi* (*s.l.*) prevalence and distribution among islands and between island and mainland sites is most likely to be due to effects of habitat fragmentation on reservoir host movements and community composition with resulting effects on pathogen persistence.

Contrary to our predictions, and results of studies on other pathogen systems [49, 50], we did not find an association between island size, or spatial isolation and *B. burgdorferi* (*s.l.*) prevalence. Within the spatial scale of our study, these predictors might be less relevant to the persistence of bird-associated genospecies that were found to dominate among island sites. In addition, the low prevalence of *B. burgdorferi* (*s.l.*) among island sites may have resulted in reduced power to detect these effects.

More generally, the low overall prevalence of *B. burgdorferi* (*s.l.*) in our system may have affected our power to detect some biological effects. For some islands, the numbers of ticks available for testing were insufficient to discriminate with confidence between *B. burgdorferi* (*s.l.*) absence and non-detection for any given year and location. Moreover, we cannot rule out persistence on islands at a lower prevalence than that found on the mainland, which could lead us to overestimate the frequency of *B. burgdorferi* (*s.l.*) extinction from islands. However, these considerations would not alter our main findings regarding the association between landscape fragmentation and decreased *B. burgdorferi* (*s.l.*) prevalence or the effect of fragmentation on the distribution and prevalence of *B. afzelii*.

Woodland cover in the UK has been reduced over the millennia from historic levels to among the lowest proportion in Europe [5]. As a result, in many areas, suitable woodland habitat for *I. ricinus* and vertebrate hosts is fragmented into patches separated by agricultural land or islands of green space within urban areas. It is possible that processes similar to those discussed above affect the persistence of *B. burgdorferi* (*s.l.*) within these fragmented mainland areas. However, a difference from our island study system and fragmented woodland surrounded by land is that ecotonal habitat surrounding fragments of woodland may be an important habitat for reservoir hosts in the latter [6]. For example, a recent study has found that ecotonal habitat and edge density affect *B. burgdorferi* (*s.l.*) prevalence in European forest fragments [51]. Surveys from England so far suggest that the dominant genospecies present are the bird-associated genospecies *B. garinii* and *B. valaisiana* with *B. afzelii* and *B. burgdorferi* (*s.s.*) being apparently absent from many sites [52–54]. Our results suggest that local extinction and limited (re)colonization opportunities for *B. afzelii* might help to explain these patterns. Policies to increase woodland area and contiguity could potentially increase the prevalence of *B. burgdorferi* (*s.l.*) by facilitating movements of reservoir host species and tick vectors between patches of suitable habitat.

Our results contrast with those from eastern North America, where fragmented woodland habitats have been associated with increased prevalence of *B. burgdorferi* [55, 56] likely due to increased densities of small mammals [57]. However, in North America, birds are still considered important in transporting infected ticks with resulting invasion of the pathogen in new areas [58]. The presence of a single generalist genospecies, *B. burgdorferi* (*s.s.*) in North America with a broad host niche that includes birds and small mammals may facilitate colonisation and persistence in fragmented habitat [59]. Our findings also disagree those of a European study which found an increased prevalence of *B. burgdorferi* (*s.l.*) in fragmented woodland but did not identify the genospecies [60]. Further studies are required to clarify the effect of habitat fragmentation on the distribution and abundance of *B. burgdorferi* (*s.l.*) in Europe and to identify commonalities and differences to North American systems.

## Conclusions

We found significantly lower prevalence of *B. burgdorferi* (*s.l.*) among island sites compared to the nearby mainland in a naturally fragmented landscape. Effects of landscape structure on pathogen prevalence was not associated with either nymph or deer abundance and was mainly a result of a significantly lower detection of pathogen presence on island sites and a lower prevalence of small mammal-associated *B. afzelii* on islands in particular. Host biology, in particular the higher mobility and less extreme population fluctuations of bird hosts compared to small mammals, is likely to play a role in explaining the wider distribution and greater prevalence of the bird-associated genospecies of *B. garinii* and the generalist genospecies *B. burgdorferi* (*s.s*.)*.* Seasonal population fluctuations of small mammal hosts may contribute to pathogen extinction, as has been seen in other disease systems [3], while limited immigration reduces opportunities for colonisation events. Pathogens maintained by highly mobile hosts such as birds which can migrate between habitat patches are predicted to be more successful in fragmented habitats.

## Additional file

Additional file 1: **Text S1.** Description of deer dung transect and deer survey methodology used to estimate deer density at Loch Lomond and the Trossachs National Park. **Text S2.** Methodology to collect nymph density data and to carry out analysis of data collected on islands and mainland sites at Loch Lomond and the Trossachs National Park. **Table S1.** Counts of selected bird species at study sites from a point transect study carried out in summer 2015. **Table S2.** Estimates of deer density at mainland and island sites (Locations of sites shown in Fig. 1) at Loch Lomond and the Trossachs National Park. Deer density was estimated using two methods described in Text S1. These methods were: counts of deer carried out on island sites only (Deer survey 2008 & 2012), an estimate of density calculated from deer dung counts along transects spaced at 200 m through each of the study sites carried out in 2015 (Dung Transects). **Table S3.** Numbers of nymphs tested for *Borrelia burgdorferi* (*sensu lato*) at each site, density of nymphs (Nymphs/10 m^2^), overall prevalence (Prev %) of *B. burgdorferi* (*s.l*.) and 95% CI, and the prevalence of each genospecies: *B. garinii* (B.g); *B. afzelii* (B.a), *Borrelia valaisiana* (B.v); *Borrelia burgdorferi* (*sensu stricto*) (B.ss) and the number of infected nymphs (n). *p(non-detect)* represents the probability of failing to detect infected ticks in a given island sample with an estimated prevalence of 0%, assuming an expected *B. burgdorferi* (*s.l*.) prevalence of 2.5% (as estimated for the mainland). Asterisks indicate cases for which the calculated probability was lower than 0.05. **Table S4.** Best model explaining questing nymphal tick variation among eleven island and 5 mainland sites in 2013 using a Poisson Generalised Linear Mixed Model. The best-fit model included vegetation type at the site of the blanket drag. Delta AICc indicates the change in AICc after removing each variable from the best-fit model. (DOCX 53 kb)
