# Supplementary material for: Landscape structure affects the prevalence and distribution of a tick-borne zoonotic pathogen
Source: Parasit Vectors. 2018 Dec 4;11:621. doi: 10.1186/s13071-018-3200-2 (PMC6278045; doi:10.1186/s13071-018-3200-2)
Supplement: Supplementary file 1 — Text S1. Description of deer dung transect and deer survey methodology used to estimate deer density at Loch Lomond and the Trossachs National Park. Text S2. Methodology to collect nymph density data and to carry out analysis of data collected on islands and mainland sites at Loch Lomond and the Trossachs National Park. Table S1. Counts of selected bird species at study sites from a point transect study carried out in summer 2015. Table S2. Estimates of deer density at mainland and island sites (Locations of sites shown in Fig. 1) at Loch Lomond and the Trossachs National Park. Deer density was estimated using two methods described in Text S1. These methods were: counts of deer carried out on island sites only (Deer survey 2008 & 2012), an estimate of density calculated from deer dung counts along transects spaced at 200 m through each of the study sites carried out in 2015 (Dung Transects). Table S3. Numbers of nymphs tested for Borrelia burgdorferi (sensu lato) at each site, density of nymphs (Nymphs/10 m2), overall prevalence (Prev %) of B. burgdorferi (s.l.) and 95% CI, and the prevalence of each genospecies: B. garinii (B.g); B. afzelii (B.a), Borrelia valaisiana (B.v); Borrelia burgdorferi (sensu stricto) (B.ss) and the number of infected nymphs (n). p(non-detect) represents the probability of failing to detect infected ticks in a given island sample with an estimated prevalence of 0%, assuming an expected B. burgdorferi (s.l.) prevalence of 2.5% (as estimated for the mainland). Asterisks indicate cases for which the calculated probability was lower than 0.05. Table S4. Best model explaining questing nymphal tick variation among eleven island and 5 mainland sites in 2013 using a Poisson Generalised Linear Mixed Model. The best-fit model included vegetation type at the site of the blanket drag. Delta AICc indicates the change in AICc after removing each variable from the best-fit model. (DOCX 53 kb) [file 13071_2018_3200_MOESM1_ESM.docx]

**Additional file 1**

**Text S1:** Description of deer dung transect and deer survey methodology used to estimate deer density at Loch Lomond and the Trossachs National Park

**Deer dung transect method**

To estimate deer density on island and mainland sites, line transects were placed in a north-south orientation the length of the study area at 200 m intervals at each site in 2015. Observations of locations of deer dung along these transects were recorded with the distance to the transect from the centre of the dung pile. Vegetation type, height and density were measured three times at 50-m intervals along the transect in the second survey and the mean of these measurements recorded. To estimate vegetation density, a sward stick with 5-cm bands on was placed in the vegetation 1 m from the observer and the proportion of the measuring stick visible recorded, and grouped into three categories; H (<0.33), M (>0.33 and <0.66), L (>0.66). The probability density function for dung detection was stratified by vegetation density to account for effects of dense vegetation on dung detection (1). The rate of dung decay was estimated by marking a representative sample of fresh dung pellets and measuring the proportion of remaining pellets at the end of the survey. Deer density was estimated by dividing the estimated dung density per km^2^ by the estimated defecation rate (21.4 pellet groups per deer per day (2), and the estimated dung decay rate (from this study) (1,3) (Table S1).

**Deer survey method**

Data from two deer surveys of the Loch Lomond Islands, carried out in March 2008 and March 2012 were obtained from Scottish National Heritage, (Jimmy Irvine, Scottish Natural Heritage personal communication) (Table S1). No deer survey data were available for the mainland sites. During the deer survey, a team of participants walked in parallel lines across each of the islands and counted all sightings of fallow deer. Boats were also positioned to count any deer which left the islands.

**Text S2:** Methodology to collect nymph density data and to carry out analysis of data collected on islands and mainland sites at Loch Lomond and the Trossachs National Park

**Testing for effects of habitat fragmentation on questing nymph density**

In 2013 twenty standardised 10 m blanket drags were carried out at each site to estimate nymph density. All sites were visited within a two week period in July 2013. At the start of each drag we measured the dominant ground vegetation type and vegetation height. Ground vegetation type within the woodlands was classified into four categories: grasses and herbaceous species, ericaceous and vaccinium species, moss species and bracken and ferns. Vegetation height was measured with a 1-m ruler. The ground temperature was measured using a RS 1360A Temperature meter (RS Components Ltd). The dominant woodland type of each site was identified by the drag location and a National Forestry Commission polygon shape file containing information on woodlands greater than 0.5 hectares (4). We categorised woodland as either predominantly broadleaved or coniferous.

To investigate if tick density was a possible factor limiting *B. burgdorferi* s.l. colonisation on islands, the total number of nymphs per 10m blanket drag was modelled using Poisson distributed errors and a log link as a function of the following explanatory variables: fragmentation class (island or mainland), vegetation type (at the drag level) and deer density from dung transects, Table S1), woodland type (broadleaved or coniferous), ground temperature at the time of sampling, and an interaction term between latitude and longitude of the site to test for spatial trends (all these variables at the site level). Site and individual drag (observation level) were entered as random effects to account for overdispersion (5). The absence of zero inflation was checked in negative binomial models during data exploration (6).

A total of 296 blanket drags carried out in 2013 with data available for all variables were included in the model to predict nymph abundance. The best fit model included ground vegetation type (Table S3), (delta AICc = 6.1). Fragmentation class (island / mainland), an index of deer density from dung transects, woodland type, ground temperature and an interaction between latitude and longitude were not maintained in the best-fit model. Significantly more ticks were found in ericaceous ground vegetation compared to grass, moss or bracken. The mean predicted number of ticks per 10m^2^ drag from the best fit model was 2.4 nymphs on ericaceous ground vegetation, compared to 1.4 nymphs on all other vegetation types.

**Table S1.** Counts of selected bird species at study sites from a point transect study carried out in summer 2015*.

| **Site** | **Blackbird** | **Blackcap** | **Chaffinch** | **Dunnock** | **Great Tit** | **Robin** | **Siskin** | **Song Thrush** | **Wren** | **Sampling stations** | **Mean birds/station** |
| --- | --- | --- | --- | --- | --- | --- | --- | --- | --- | --- | --- |
| BA | 0 | 6 | 2 | 0 | 11 | 11 | 0 | 3 | 25 | 8 | 7.3 |
| MA | 7 | 11 | 9 | 0 | 3 | 14 | 0 | 6 | 18 | 9 | 7.6 |
| SA | 4 | 6 | 4 | 0 | 5 | 6 | 2 | 1 | 9 | 8 | 4.6 |
| BU | 1 | 0 | 3 | 0 | 5 | 1 | 0 | 0 | 6 | 2 | 8 |
| CA | 4 | 1 | 8 | 0 | 7 | 11 | 0 | 0 | 24 | 10 | 5.5 |
| CE | 5 | 1 | 2 | 1 | 2 | 6 | 0 | 0 | 13 | 4 | 7.5 |
| CL | 0 | 0 | 10 | 0 | 7 | 4 | 0 | 0 | 10 | 3 | 10.3 |
| CO | 0 | 4 | 17 | 0 | 5 | 21 | 0 | 0 | 19 | 8 | 8.3 |
| CR | 0 | 0 | 7 | 0 | 4 | 4 | 0 | 0 | 9 | 5 | 4.8 |
| FA | 2 | 5 | 13 | 2 | 3 | 5 | 2 | 1 | 24 | 7 | 8.1 |
| LO | 2 | 1 | 14 | 0 | 7 | 11 | 1 | 0 | 22 | 8 | 7.3 |
| MO | 0 | 6 | 3 | 0 | 2 | 1 | 0 | 0 | 7 | 8 | 2.4 |
| MU | 7 | 0 | 8 | 2 | 6 | 8 | 0 | 0 | 25 | 11 | 5.1 |
| TA | 1 | 2 | 7 | 0 | 7 | 6 | 0 | 0 | 16 | 8 | 4.9 |
| TO | 1 | 0 | 1 | 0 | 1 | 1 | 0 | 0 | 11 | 3 | 5 |

*A point transect sampling method was used to record birds with sampling locations separated by 250 m within each site (7). All birds seen or heard were counted, regardless of their behaviour or age. Birds were counted for 15 minutes at each sampling location. In order to compare the abundance of birds most relevant as tick hosts between sites, only data for the nine bird species considered to be the most significant tick hosts in Scotland were used (8,9) .

**Table S2.** Estimates of deer density at mainland and island sites (Locations of sites shown in Figure 1) at Loch Lomond and the Trossachs National Park. Deer density was estimated using two methods described in Text S1. These methods were; counts of deer carried out on island sites only (Deer survey 2008 & 2012), an estimate of density calculated from deer dung counts along transects spaced at 200m through each of the study sites carried out in 2015 (Dung Transects).

| **Site** | **Location** | **Deer Survey 2008** | **Deer Survey 2012** | **Dung Transects** |
| --- | --- | --- | --- | --- |
| BA | Mainland | NA | NA | 51.5 |
| BW | Mainland | NA | NA | 22.4 |
| KN | Mainland | NA | NA | 22.7 |
| MA | Mainland | NA | NA | 29.8 |
| SA | Mainland | NA | NA | 17.0 |
| BU | Island | 0.0 | 0.0 | 22.2 |
| CA | Island | 101.9 | 35.8 | 24.6 |
| CE | Island | 193.0 | 122.8 | 20.9 |
| CL | Island | 0.0 | 0.0 | 54.2 |
| CO | Island | 0.0 | 4.8 | 2.8 |
| CR | Island | 10.6 | 45.9 | 54.7 |
| FA | Island | 228.4 | 33.3 | 74.7 |
| LO | Island | 46.6 | 39.9 | 70.6 |
| MO | Island | 30.7 | 21.9 | 26.7 |
| MU | Island | 0.0 | 0.0 | 16.9 |
| TA | Island | 42.9 | 33.3 | 69.2 |
| TO | Island | 13.3 | 40.0 | 20.6 |

**Table S3.** Numbers of nymphs tested for *Borrelia burgdorferi* sensu lato at each site, density of nymphs (Nymphs/10m^2^), overall prevalence (Prev %) of *B. burgdorferi* s.l. and 95% C.I., and the prevalence of each genospecies, B.g = *B. garinii*, B.a = *B. afzelii*, B.v = *Borrelia valaisiana,* B.ss = *Borrelia burgdorferi* sensu stricto and the number of infected nymphs (n). *p(non-detect)* represents the probability of failing to detect infected ticks in a given island sample with an estimated prevalence of 0%, assuming an expected *B. burgdorferi* s.l. prevalence of 2.5% (as estimated for the mainland). Asterisks indicate cases for which the calculated probability was lower than 0.05.

| **Site** | **Type** | **Year** | **Nymphs tested** | **Nymphs/ 10 m^2^ (SD)** | **Prev % (95% CI)** | ***B.g*% (n)** | **B.a% (n)** | **B.v% (n)** | **B.ss% (n)** | ***p(non-detect)*** |
| --- | --- | --- | --- | --- | --- | --- | --- | --- | --- | --- |
| BA | Mainland | 2013 | 194 | 2.8 (2.3) | 2.6  (0.8-5.9) | 0.5 (1) | 0.5 (1) | 0.5 (1) | 1.0 (2) | NA |
|  |  | 2015 | 200 | 9.1 (4.1) | 4.5 (2.4-8.3) | 0.5 (1) | 0.0 | 0.0 | 4.0 (8) | NA |
| BW | Mainland | 2013 | 184 | 1.3 (1.7) | 0 .0 (0.0-2.0) | 0.0 | 0.0 | 0.0 | 0.0 | NA |
| KN | Mainland | 2011 | 149 | 7.6 (6.0) | 2.0 (0.4-5.8) | 0.0 | 2.0 (3) | 0.0 | 0.0 | NA |
|  |  | 2013 | 197 | 1.1 (1.1) | 1.0 (0.1-3.6) | 0.5 (1) | 0.5 (1) | 0.0 | 0.0 | NA |
| LU | Mainland | 2011 | 109 | 3.8 (3.6) | 4.6 (1.5-10.4) | 1.8 (2) | 2.8 (3) | 0.0 | 0.0 | NA |
|  |  | 2013 | 116 | 1.6 (1.5) | 2.6 (0.5-7.4) | 2.6 (3) | 0.0 | 0.0 | 0.0 | NA |
| MA | Mainland | 2011 | 146 | 3.5 (3.1) | 2.7 (0.7-6.9) | 2.7 (4) | 0.0 | 0.0 | 0.0 | NA |
|  |  | 2015 | 184 | 6.1 (4.0) | 2.2 (0.8-5.5) | 2.2 (4) | 0.0 | 0.0 | 0.0 | NA |
| SA | Mainland | 2013 | 212 | 4.5 (3.1) | 3.3 (1.3-6.7) | 0.0 | 0.5 (1) | 0.5 (1) | 2.4 (5) | NA |
|  |  | 2015 | 200 | 7.5 (6.1) | 2.5 (1.1-5.7) | 1.5 (3) | 0.0 | 1.0 (2) | 0.0 | NA |
| BU | Island | 2011 | 130 | 2.9 (1.9) | 3.8 (1.3-8.7) | 3.8 (5) | 0.0 | 0.0 | 0.0 | NA |
|  |  | 2013 | 197 | 3.8 (4.8) | 3.6 (1.5-7.3) | 2.5 (5) | 0.0 | 1.0 (2) | 0.0 | NA |
|  |  | 2015 | 149 | 5.9 (3.7) | 0.0 (0-2.5) | 0.0 | 0.0 | 0.0 | 0.0 | 0.023* |
| CA | Island | 2011 | 149 | 17.7 (10.2) | 0 (0.0-2.4) | 0.0 | 0.0 | 0.0 | 0.0 | 0.023* |
|  |  | 2013 | 205 | 5.6 (4.5) | 0.0 (0-1.8) | 0.0 | 0.0 | 0.0 | 0.0 | 0.006* |
|  |  | 2015 | 185 | 5.9 (3.1) | 0.0 (0.0-2.0) | 0.0 | 0.0 | 0.0 | 0.0 | 0.009* |
| CE | Island | 2013 | 116 | 0.1 (0.3) | 0.0 (0.0-3.1) | 0.0 | 0.0 | 0.0 | 0.0 | 0.053 |
|  |  | 2015 | 33 | 1.7 (1.2) | 21.2 (10.7 - 37.8) | 21.2 (7) | 0.0 | 0.0 | 0.0 | NA |
| CL | Island | 2015 | 114 | 4.5 (3.7) | 2.6 (0.9 - 7.5) | 2.6 (3) | 0.0 | 0.0 | 0.0 | NA |
| CO | Island | 2011 | 111 | 1.4 (2.0) | 0.0 (0.0-3.3) | 0.0 | 0.0 | 0.0 | 0.0 | 0.060 |
|  |  | 2013 | 189 | 1.3 (1.4) | 0(0.0-1.9) | 0.0 | 0.0 | 0.0 | 0.0 | 0.008* |
|  |  | 2015 | 79 | 3.9 (2.7) | 5.1 (2.0 - 12.3) | 0.0 | 0.0 | 0.0 | 5.1 (4) | NA |
| CR | Island | 2011 | 102 | 5.5 (4.2) | 2.0 (0.2-6.9) | 1.0 (1) | 1.0 (1) | 0.0 | 0.0 | NA |
|  |  | 2013 | 210 | 10.2 (15.4) | 0.0 (0.0-1.7) | 0.0 | 0.0 | 0.0 | 0.0 | 0.005* |
|  |  | 2015 | 200 | 27.1 (17.3) | 1.5 (0.5 - 4.3) | 0.0 | 0.0 | 0.0 | 1.5 (3) | NA |
| FA | Island | 2013 | 172 | 1.4 (2.6) | 0.0 (0.0-2.1) | 0.0 | 0.0 | 0.0 | 0.0 | 0.013* |
|  |  | 2015 | 64 | 1.6 (1.5) | 3.1 (0.9 - 10.7) | 0.0 | 0.0 | 0.0 | 3.1 (2) | NA |
| LO | Island | 2011 | 141 | 37.8 (28.8) | 0.0 (0.0-2.6) | 0.0 | 0.0 | 0.0 | 0.0 | 0.028* |
|  |  | 2013 | 185 | 7.8 (5.7) | 0.0 (0.0-2.0) | 0.0 | 0.0 | 0.0 | 0.0 | 0.009* |
|  |  | 2015 | 200 | 21.6 (27.7) | 0.5 (0.1 - 2.8) | 0.0 | 0.0 | 0.0 | 0.5 (1) | NA |
| MO | Island | 2013 | 209 | 3.6 (3.7) | 0.0 (0.0-1.7) | 0.0 | 0.0 | 0.0 | 0.0 | 0.005* |
|  |  | 2015 | 198 | 22.6 (14.0) | 0.0 (0.0 - 1.9) | 0.0 | 0.0 | 0.0 | 0.0 | 0.007* |
| MU | Island | 2011 | 146 | 2.8 (2.1) | 3.4 (1.1-7.8) | 2.7 (4) | 0.0 | 0.7 (1) | 0.0 | NA |
|  |  | 2013 | 66 | 0.4 (0.5) | 0.0 (0.0-5.4) | 0.0 | 0.0 | 0.0 | 0.0 | 0.188 |
|  |  | 2015 | 22 | 0.8 (0.8) | 0.0 (0.0 - 14.9) | 0.0 | 0.0 | 0.0 | 0.0 | 0.573 |
| TA | Island | 2011 | 193 | 3.3 (3.1) | 0.5 (0.0-2.9) | 0.0 | 0.0 | 0.5 (1) | 0.0 | NA |
|  |  | 2013 | 187 | 0.7 (0.9) | 1.1 (0.1-3.8) | 0.5 (1) | 0.0 | 0.5 (1) | 0.0 | NA |
|  |  | 2015 | 188 | 4.1 (2.5) | 1.1 (0.3 - 3.8) | 1.1 (2) | 0.0 | 0.0 | 0.0 | NA |
| TO | Island | 2011 | 164 | 42.9 (25.7) | 0 .0 (0.0-2.2) | 0.0 | 0.0 | 0.0 | 0.0 | 0.016* |
|  |  | 2013 | 229 | 3.7 (2.2) | 0(0.0-1.6) | 0.0 | 0.0 | 0.0 | 0.0 | 0.003* |
|  |  | 2015 | 143 | 6.6 (6.2) | 0.0 (0.0 - 2.6) | 0.0 | 0.0 | 0.0 | 0.0 | 0.027* |

**Table S4.** Best model explaining questing nymphal tick variation among eleven island and 5 mainland sites in 2013 using a Poisson Generalised Linear Mixed Model. The best fit model included vegetation type at the site of the blanket drag. Delta AICc indicates the change in AICc after removing each variable from the best fit model.

| **Fixed Effects** | **Mean (Estd)** | **SE** | **p value** | **Delta AICc** |
| --- | --- | --- | --- | --- |
| Intercept | 0.31 | 0.28 | 0.26 | - |
| Vegetation type (Bracken) | - | - | - | 6.1 |
| Ericaceous vegetation | 0.58 | 0.18 | 0.001 | - |
| Grass vegetation | 0.0067 | 0.15 | 0.97 | - |
| Moss vegetation | 0.027 | 0.21 | 0.90 | - |

**References**

1. Marques FFC, Buckland ST, Goffin D, Dixon CE, Borchers DL, Mayle BA, et al. Estimating deer abundance from line transect surveys of dung: Sika deer in southern Scotland. J Appl Ecol. 2001;38(2):349–63.

2. Mayle B, Peace A, Gill R. How Many Deer? A guide to estimating deer population size. Edinburgh: Forestry Commission; 1999. 96 p.

3. Laing SE, Buckland ST, Burn RW, Lambie D, Amphlett A. Dung and nest surveys: Estimating decay rates. J Appl Ecol. 2003;40(6):1102–11.

4. Whitton E. Forestry Commission [Internet]. National Forest Inventory Great Britain 2012 shapefile. http://www.forestry.gov.uk/datadownload. 2013 [cited 2013 May 15]. Available from: http://www.forestry.gov.uk/datadownload

5. Harrison X A. A comparison of observation-level random effect and Beta-Binomial models for modelling overdispersion in Binomial data in ecology & evolution. PeerJ. 2015;3:e1114.

6. Zuur AF, Ieno EN, Walker NJ, Saveliev AA, Smith GM. Mixed Effects Models and Extensions in Ecology with R. New York, USA: Springer; 2009.

7. Bibby C, Jones M, Marsden S. Expedition field techniques: Bird Surveys. Cambridge: Geography Outdoors; 1998.

8. James MC, Furness RW, Bowman AS, Forbes KJ, Gilbert L. The importance of passerine birds as tick hosts and in the transmission of *Borrelia burgdorferi*, the agent of Lyme disease: a case study from Scotland. Ibis (Lond 1859). 2011 Apr 17;153(2):293–302.

9. Millins C. Ecological drivers of a vector borne pathogen: Distribution and abundance of *Borrelia burgdorferi* sensu lato and its vector *Ixodes ricinus* in Scotland. University of Glasgow PhD thesis; 2016.
